# Molecular Mechanism of Resistance to *Alternaria alternata* Apple Pathotype in Apple by Alternative Splicing of Transcription Factor *MdMYB6-like*

**DOI:** 10.3390/ijms25084353

**Published:** 2024-04-15

**Authors:** Xianqi Zeng, Chao Wu, Lulu Zhang, Liming Lan, Weihong Fu, Sanhong Wang

**Affiliations:** College of Horticulture, Nanjing Agricultural University, Nanjing 210095, China; 2021104032@stu.njau.edu.cn (X.Z.); 2019204014@stu.njau.edu.cn (C.W.); 2022104017@stu.njau.edu.cn (L.Z.); 2021204011@stu.njau.edu.cn (L.L.); 2020204012@stu.njau.edu.cn (W.F.)

**Keywords:** *Alternaria alternata* apple pathotype, alternative splicing, apple, biotic stress, R2R3-MYB

## Abstract

As a fruit tree with great economic value, apple is widely cultivated in China. However, apple leaf spot disease causes significant damage to apple quality and economic value. In our study, we found that *MdMYB6-like* is a transcription factor without auto-activation activity and with three alternative spliced variants. Among them, *MdMYB6-like-β* responded positively to the pathogen infection. Overexpression of *MdMYB6-like-β* increased the lignin content of leaves and improved the pathogenic resistance of apple flesh callus. In addition, all three alternative spliced variants of *MdMYB6-like* could bind to the promoter of *MdBGLU H*. Therefore, we believe that *MdMYB6-like* plays an important role in the infection process of the pathogen and lays a solid foundation for breeding disease-resistant cultivars of apple in the future.

## 1. Introduction

Apple (*Malus domestica*) is one of the most popular fruits in the world, widely grown around the world because of its pleasing flavor and rich nutritional value, as well as its ease of cultivation and harvesting [1]. Currently, apple cultivation area and output in China are among the highest in the world, making it one of the most important cash crops and fruits in the country.

Currently, apple leaf diseases are the main cause of serious impacts on yield and quality in the apple production, resulting in significant production and economic losses [2]. Apple spotted leaf is a fungal disease caused by *Alternaria alternata* apple pathotype (AAAP), which was first reported in the southern part of Iwate Prefecture, Japan, in the early 19th century [3]. This disease occurs in most apple growing areas of the world, particularly in East Asia [4,5]. The disease mainly affects the young parts of the plant, including leaves, branches and fruit. In the early stages of the disease, sub-circular spots appear on the leaves, and, as the disease progresses, the spots increase in size and the leaves gradually become perforated, dry up and eventually fall off. It has a serious negative impact on the growth of the tree, which ultimately affects fruit yield and quality [6,7]. At present, the main methods of controlling fungal diseases in orchards are chemical control and physical control. These methods not only fail to provide a fundamental solution to the disease problem but also reduce the resistance of fruit trees to pathogenic fungi. They cause serious pollution of the environment and are even harmful to human health [8,9]. Therefore, the discovery of disease-resistant genes, the selection of disease-resistant apple resources and the selecting and breeding of disease-resistant cultivars are the most effective and safest options for controlling spotted leaf disease.

In fact, plant breeders have been using resistance genes to control plant diseases for nearly a century. In Arabidopsis, several families of transcription factors with functional domains, such as ERF, bZIP/HD-ZIP, WRKY and MYB, are involved in the regulation of defense responses [10]. The MYB transcription factor family is one of the largest transcription factor families and is grouped into four subfamilies, R1, R2R3, R1R2R3 and 4R [11]. There is increasing evidence that R2R3-MYB transcription factors are involved in the response to a variety of abiotic and biotic stresses. In Arabidopsis, AtMYB96 and AtMYB44 increased resistance to Pseudomonas syringae by mediating the salicylic acid-signaling pathway in response to pathogen infection and enhancing the pathogenesis-related (PR) gene expression, respectively [12,13]. Heterologous overexpression of sweet cherry *PacMYBA* in Arabidopsis thaliana improves its resistance to *Pseudomonas syringe pv. tomato (Pst) DC3000* [14]. Overexpression of *PnMYB2* from Panax notoginseng in tobacco can improve resistance to *F. solani* [15]. In tomato, heterologous overexpression of the rice *OsMYB4* gene increases the resistance of transgenic plants to the *tomato mosaic virus* [16]. Transgenic tobacco plants overexpressing tomato *SpMYB* had improved salt and drought stress tolerance compared to wild-type plants and showed significantly improved resistance to Alternaria alternate [17]. The Chinese wild grape (*Vitis davidii*) R2R3-MYB transcription factor *VdMYB1* can activate the *STS2* gene expression and positively regulate the defense response to increase resveratrol levels in leaves, thereby enhancing resistance to pathogenic fungi [18].

Alternative splicing (AS) is the creation of multiple mRNAs from the same gene by variable splice site selection during pre-mRNA processing. This process results in the production of different protein types, thereby increasing the functional diversity encoded by the gene [19]. Typical AS events include skipped exon (SE), retained intron (RI), alternative 5′ splice site (A5SS), alternative 3′ splice site (A3SS), mutually exclusive exon (MXE), alternative first exon (AFE) and alternative last exon (ALE) [20]. There is increasing evidence that AS plays a key regulatory role in regulating gene expression during development and in response to environmental signals [21,22,23]. Former authors analyzed plants grown under normal growth conditions and concluded that more than 60% of intron-containing genes undergo alternative splicing [24,25]. Previous studies have shown that AS occurs in many plants in response to the environment, as well as in the regulation of dormancy or senescence and in the regulation of the accumulation of secondary metabolites, for example, *Arabidopsis thaliana*, grape, chrysanthemum, ‘Dangshansuli’ pear and poplar [26,27,28,29,30]. However, the phenomenon of AS occurring in plants in response to biotic stress is poorly understood. There are differences in the incidence and severity of apple leaf spot disease between different apple cultivars, with the *Alternaria alternata* apple pathotype infecting nearly 85% of the leaves of susceptible cultivars but less than 1% of the leaves of resistant cultivars [8,31]. For example, apple cultivars such as ‘Jonathan’, ‘Gala’ and ‘Jiguan’ are resistant to the disease, but ‘Starking Delicious’, ‘Indo’ and ‘Orin’ are highly susceptible [3].

Research on the resistance mechanism of apple leaf spot disease, the identification of disease-resistant genes in apples and breeding of disease-resistant cultivars have become hotspots in apple biology research. In this study, we identified *MdMYB6-like* as a member of the R2R3-MYB transcription factor family, which regulates the phenylpropanoid metabolic pathway and undergoes alternative splicing in the ‘Starking Delicious’ cultivar. Exploring the function of its alternative splice variants revealed a positive effect on apple resistance to AAAP infection. ‘Starking Delicious’ apple is a major cultivar in China and is highly sensitive to AAAP, which has led to enormous damage to apple production. This study holds important significance for further resistance breeding efforts.

## 2. Results

### 2.1. Three Alternative Spliced Variants of MdMYB6-like in Apple

**Figure 1:**
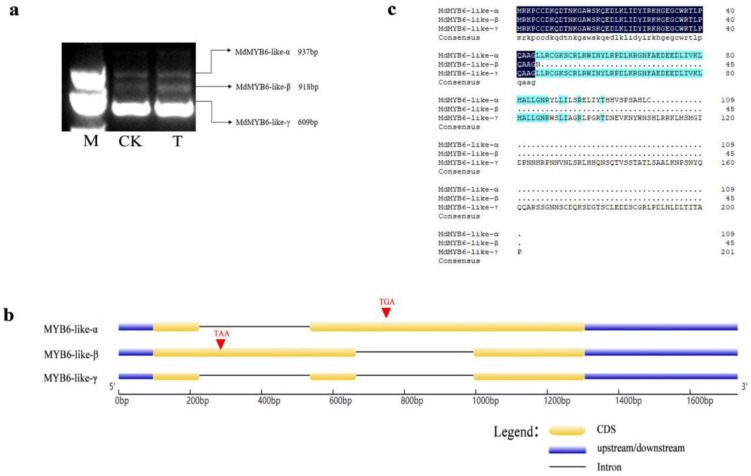
Cloning and structure analysis of *MdMYB6-like* and its novel variants: (**a**) the three alternative spliced variants of *MdMYB6-like* confirmed by RT-PCR using ‘Starking Delicious’ cDNA; (**b**) diagrammatic representation of the genomic and protein structure of the three alternative spliced variants. The red triangle indicates a premature termination codon (PTC); (**c**) amino acid sequence analysis of the three alternative spliced variants.

We found that MdMYB6-like undergoes alternative spliced events. Through previous studies in our laboratory and analysis of RNA sequencing (RNA-seq) data, we found that MdMYB6-like alternative splicing events occur. As a result, we used cDNA reverse transcribed from RNA extracted from both infected and healthy leaves affected by leaf spot disease as a template for RT-PCR, using primers specific for *MdMYB6-like*. Three products of 609 bp, 918 bp and 937 bp were cloned. We named the three alternative spliced variants *MdMYB6-like-α*, *MdMYB6-like-β* and *MdMYB6-like-γ*, respectively (Figure 1a). According to structural analysis and comparison with the apple genome, *MdMYB6-like-α* and *MdMYB6-like-β* retained intron 2 and intron 1, respectively, during pre-RNA processing (Figure 1b). This suggests that the alternative splicing event type is retained intron (RI). Sequence analysis showed that the retention of the intron resulted in a move forward of the termination codon for both variants (Figure S1). This ultimately leads to different degrees of early translation termination for both variants, except for *MdMYB6-like-γ* (Figure 1c).

### 2.2. The Evolutionary Analysis of MdMYB6-like in Apple

**Figure 2:**
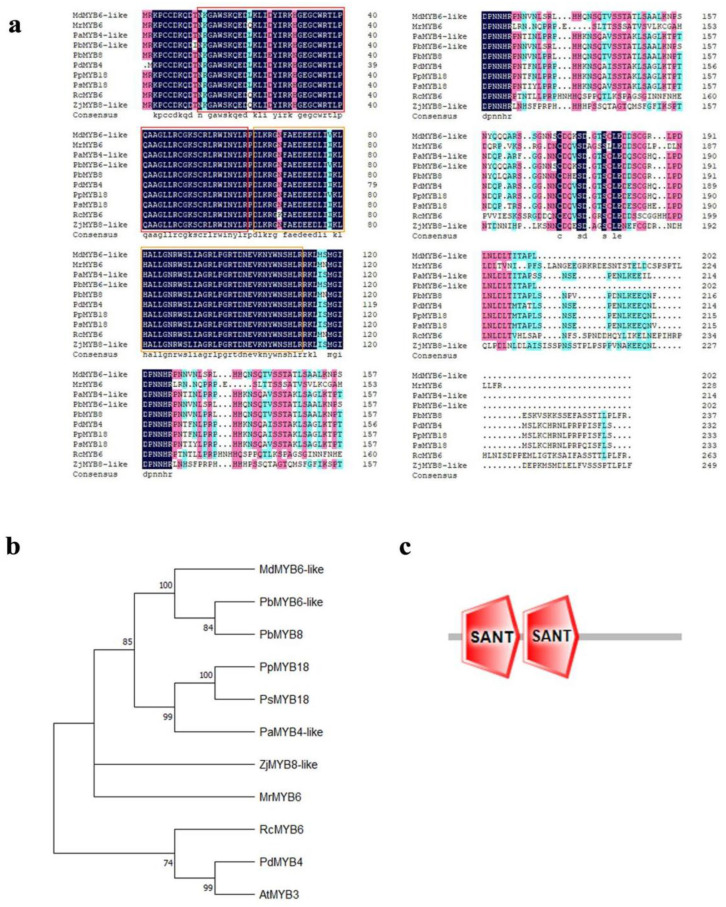
Amino acid sequence alignment and phylogenetic tree construction of *MdMYB6-like-α*, *MdMYB6-like-β*, *MdMYB6-like-γ* and other highly homologous MYB TFs. (**a**) Amino acid sequence comparison between *MdMYB6-like-α*, *MdMYB6-like-β*, *MdMYB6-like-γ* and ten R2R3-MYB TFs. The following amino acid sequences were retrieved from the GenBank database: *MdMYB6-like* (sequence ID: XP_017192216.1), *MrMYB6* (sequence ID: KAB1209240.1), *PaMYB4-like* (sequence ID: XP_021824312.1), *PbMYB6-like* (sequence ID: XP_018502946.2), *PbMYB8* (sequence ID: XP_009357784.1), *PdMYB4* (sequence ID: BBG94536.1), *PpMYB18* (sequence ID: ALO81021.1), *PsMYB18* (sequence ID: QGQ60117.1), *RcMYB6* (sequence ID: XP_024196862.1), *ZjMYB8-like* (sequence ID: XP_048318055.2). The red and orange boxes represent the R1 and R2 structural domains, respectively. (**b**) Phylogenetic tree of *MdMYB6-like-α*, *MdMYB6-like-β*, *MdMYB6-like-γ* and their homologs in different plant species. The tree is drawn proportionally. Numbers next to the node are bootstrap values from 1000 replications. (**c**) Protein structures of three alternative spliced variants generated with SMART.

The blastp function of the NCBI website was used to find MYB transcription factors highly homologous to *MdMYB6-like* in other plants, facilitating protein sequence comparison. The results showed that the selected MYB transcription factors all contain two conserved R2 and R3 MYB structural domains, which is a characteristic of the R2R3-MYB transcription factor (Figure 2a). The constructed phylogenetic tree showed that *MdMYB6-like* was closely related to *PbMYB8* in pear (Figure 2b). Additionally, previous studies indicated that *PbMYB8* is involved in the expression of lignin metabolism pathway in pear. Therefore, we presumed that *MdMYB6-like* also plays an important role in the lignin metabolic pathway [32]. The predicted protein structure indicates that *MdMYB6-like* contains two conserved structural domains, indicating its membership in the SANT supergene family (Figure 2c). The SANT domain is involved in histone acetylation and deacetylation and is associated with chromatin remodeling [33].

### 2.3. The Expression of MdMYB6-like in Response to Alternaria alternata Apple Pathotype Infection

**Figure 3:**
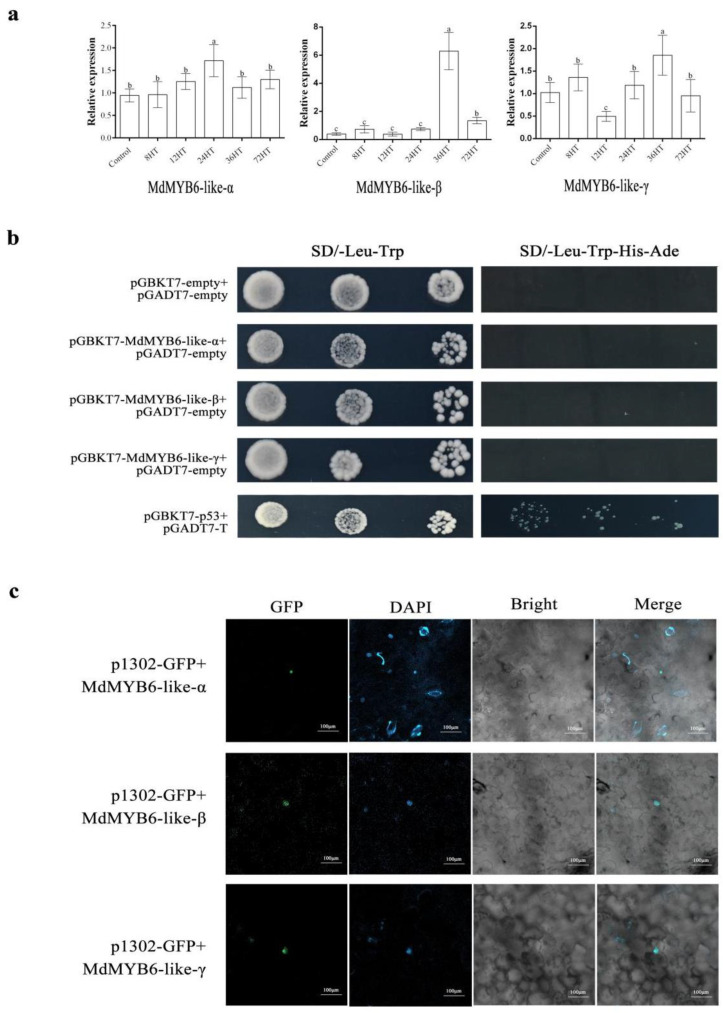
The expression levels after infection by AAAP and identification of transcription factor properties of MdMYB6-like and its alternative spliced variants: (**a**) differential expression of *MdMYB6-like* and its alternative spliced variants in leaves after different times (0, 12, 24, 36, 72 h) of infection. Error bars represent the SDs from three biological replicates. Lowercase letters represent significant differences at *p* < 0.05 (Tukey’s HSD test). (**b**) Yeast two-hybrid assays of auto-activation activity identification of MdMYB6-like and its alternative spliced variants. Co-transformation of empty pGADT7 and pGBKT7 vector was used as a negative control, and co-transformation of pGBKT7-p53 and pGADT7-T vector was used as a positive control. Each co-transformation was diluted ten times. (**c**) Subcellular localization of GFP-MdMYB6-like-α, GFP-MdMYB6-like-β and GFP- MdMYB6-like-γ in leaf cells of *N. benthamiana.* The constructed plasmids for the assay were transferred into Agrobacterium tumefaciens GV3101 and transiently transfected into *N. benthamiana* leaves. DAPI was used to stain the nucleus prior to observations of GFP fluorescence. GFP, DAPI, bright-field, and merged images are presented. All bars = 100 μm.

In order to investigate the role of *MdMYB6-like-α*, *MdMYB6-like-β* and *MdMYB6-like-γ* in the development of apple diseases, we performed inoculation experiments with the AAAP pathogen (Figure S2a) and determination of lignin content (Figure S2b) on ‘Starking Delicious’ and ‘Jonathan’ leaves of the same age in the same week, respectively. Then we collected and tested leaf samples from different inoculation times. As a result, we found that all three variants increased in ‘Starking Delicious’ as the disease intensified, with *MdMYB6-like-β* showing a highly significant increase at 36 h post-inoculation with the pathogen (Figure 3a), suggesting that its expression product may play a role in disease development.

### 2.4. Subcellular Localization of MdMYB6-like

An online tool (http://www.csbio.sjtu.edu.cn/bioinf/Cell-PLoc-2/, accessed on 12 April 2023) was used to predict the localization of *MdMYB6-like* in plant cells.

*MdMYB6-like-α*, *MdMYB6-like-β* and *MdMYB6-like-γ* were constructed in the pCAMBIA1302-GFP vector and transferred into *Agrobacterium* strain GV3101. The *Agrobacterium* was resuspended in infiltration buffer and then injected into *N. benthamiana* leaves for cultivation. Subcellular localization analyses were performed on DAPI-stained *N. benthamiana* leaves to determine whether the AS events in *MdMYB6-like* affect the distribution of the encoded proteins. The results showed that AS events did not change the localization of the encoded variant proteins. The GFP signals of *MdMYB6-like-α*, *MdMYB6-like-β* and *MdMYB6-like-β* were all located in the nucleus (Figure 3b).

### 2.5. MdMYB6-like Auto-Activation Activity Identification

*First*, we validated the auto-activation activity of *MdMYB6-like-α*, *MdMYB6-like-β* and *MdMYB6-like-γ* using yeast two-hybridization. We co-transformed BD-*MdMYB6-like-α*, BD-*MdMYB6-like-γ* and BD-*MdMYB6-like-β* into the yeast strain AH109 together with the empty pGADT7 vector. Co-transformation of empty pGADT7 and pGBKT7 vectors was used as a negative control, and co-transformation of pGBKT7-p53 and pGADT7-T vectors was used as a positive control. The results showed that the three variants could grow normally on SD/-Trp/-Leu medium but not on SD-Leu/-Trp/-His/-Ade medium, so we concluded that none of the three variants had auto-activation activity (Figure 3c).

### 2.6. Validation of Interactions between MdMYB6-like Variants

To determine whether the c-terminal deletion of *MdMYB6-like* could cause the two variants to bind to each other, we performed a validation using the yeast two-hybrid assay. We constructed the three variants on pGBKT7 and pGADT7, respectively, and then co-transformed them into the yeast strain AH109 in different combinations. The control setup was the same as in the auto-activation activity experiment. As a result, we found that during normal growth, the various variants do not bind to each other or to themselves to form homodimers to regulate growth and development (Figure S3).

### 2.7. MdMYB6-like-β Can Impact the Lignin Content of Apple Leaves

**Figure 4:**
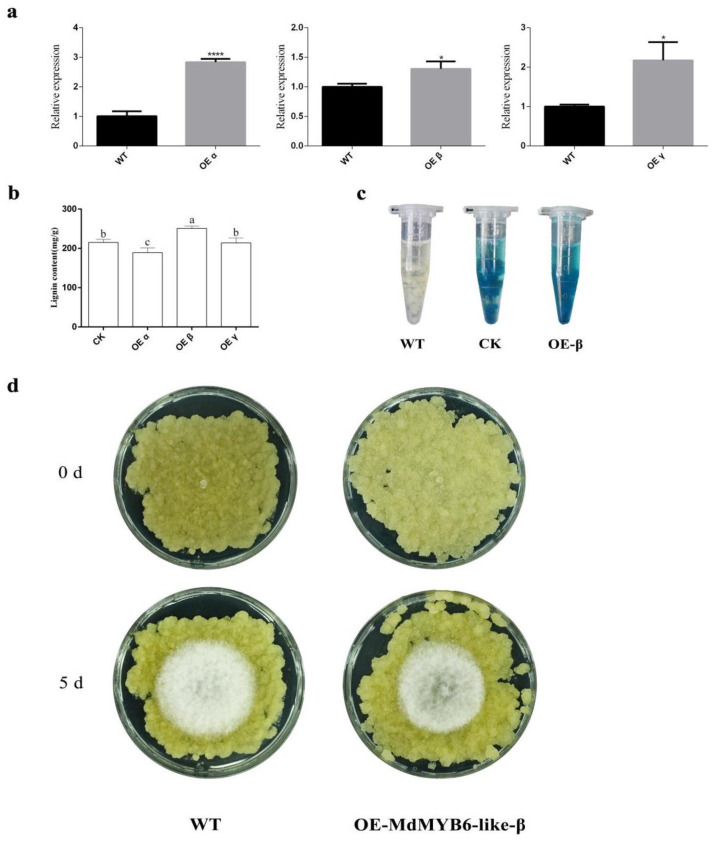
Effects of transient overexpression and stable overexpression on apple materials. (**a**) RT-qPCR analysis of *MdMYB6-like* and its alternative spliced variants in the transient overexpression of ‘Gala’ apple leaves. Error bars represent the SDs from three biological replicates. The level of significance is indicated by *, denoting significant differences at *p* < 0.05, The level of significance is indicated by ****, denoting significant differences at *p* < 0.0001; (**b**) lignin content analysis of ‘Gala’ apple leaves following transient overexpression of the three alternatively spliced variants. Error bars represent the SDs from three biological replicates. Lowercase letters represent significant differences at *p* < 0.05; (**c**) wild-type, overexpressing empty pCAMBIA2300 vector and overexpressing pCAMBIA2300-*MdMYB6-like-β* apple flesh callus were placed into 1.5 mL centrifuge tubes for GUS staining, respectively. Blue color means that the overexpression vector was successfully transferred into apple flesh callus; (**d**) the inoculation AAAP experiment on normal-growing apple flesh callus. The white round spots represent the area of fungal infection after 5 d and indicate the amount of mycelia.

In order to investigate the function of *MdMYB6-like* in the disease resistance of apple leaves, we constructed the three variants into the overexpression vector pCAMBIA2300 and subjected ‘Gala’ apple leaves to transient overexpression experiments. We used overexpression of the empty pCAMBIA2300 vector as a control in the experiment. We immersed ‘Gala’ apple leaves in resuspended *Agrobacterium* with infiltration buffer and created a vacuum environment to promote infection. The transient overexpression experiments increased the expression levels of the various variants in apple leaves, respectively, as shown by RT-qPCR experiments (Figure 4a). After that, we analyzed the lignin content of the overexpressed apple leaves. The results showed that overexpression of *MdMYB6-like-β* variants significantly increased the lignin content in leaves (Figure 4b).

We analyzed the expression of the genes encoding enzymes involved in the lignin biosynthetic pathway using RT-qPCR experiments. The results showed that overexpression of *MdMYB6-like-α*, *MdMYB6-like-β* and *MdMYB6-like-γ* down-regulated the expression of some lignin synthesis-related genes, such as *MdPAL*, *MdC3H*, *MdCAD* and *MdCOMT*, but up-regulated the expression of the coumarin-synthesizing gene *MdBGLU42*. The expression of other lignin synthesis-related genes varied due to the overexpression of different variants. Specifically, the expression of *MdLac3* significantly increased after the overexpression of *MdMYB6-like-β*, and it was much higher than both the control and the other two variants (Figure S4). This suggests that the overexpression of the *MdMYB6-like-β* variant is an important gene that can significantly increase the lignin content in leaves.

Among plant metabolites, lignin, a phenolic compound, thickens the middle lamella and the secondary cell walls of plants against fungal infection. This not only helps to resist the invasion of pathogenic fungi in terms of physical defenses, but also the catalytic activities of lignin biosynthetic enzymes also contribute to the accumulation of other defense compounds [34]. Therefore, in the following experiments, we focused on the *MdMYB6-like-β* variant.

### 2.8. Stable Overexpression of MdMYB6-like-β Isoforms Increases the Resistance of Apple Flesh Callus to Pathogens

In the previous results, it was shown that overexpression of *MdMYB6-like-β* did indeed increase the lignin content of apple leaves. To determine whether *MdMYB6-like-β* actually increases apple resistance to pathogen invasion, we performed experiments with apple flesh callus overexpressing the *MdMYB6-like-β* variant. The β-glucuronidase (GUS) staining was used to test whether the GUS gene could be expressed in apple flesh callus, enabling the selection of positive and stably transformed apple flesh callus [35] (Figure 4c). We used the apple flesh callus successfully overexpressing empty pCAMBIA2300 as the control to perform inoculation experiments with AAAP, together with stably transformed overexpression of the *MdMYB6-like-β* variant. The results showed that the patch areas of apple flesh callus with the stably transformed overexpression of the *MdMYB6-like-β* variant were significantly smaller than those of the empty control check after five days of inoculation (Figure 4d).

In this regard, combined with the comprehensive analysis of the results of the previous experiments, this finding leads us to believe that the *MdMYB6-like-β* variant plays a key role in the resistance of apples to pathogen fungi invasion by inducing an increase in lignin content and thickening of the secondary cell wall.

### 2.9. MdMYB6-like Can Bind to the Promoter of MdBGLU H

**Figure 5:**
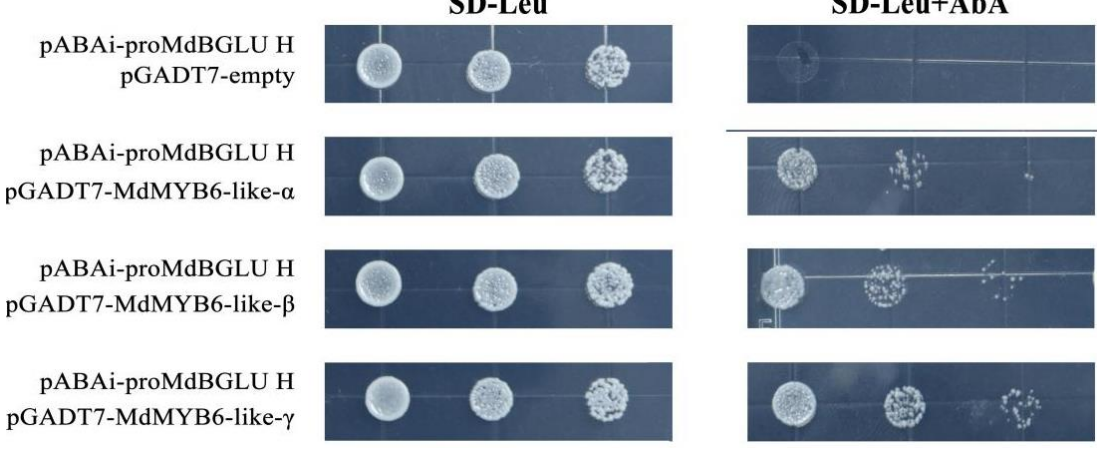
*MdMYB6-like* regulation of other genes. Yeast one-hybrid assays were conducted on SD-Leu medium, supplemented with appropriate AbA concentrations (0 or 100 ng/mL) to verify DNA–protein interactions, with each co-transformation diluted 10 times. Transformation of empty pGADT7 vector with promoter of the *MdBGLU H* was used as a negative control.

Previous studies have shown that R2R3-MYB transcription factors can regulate the expression of downstream genes by directly binding to AC-I, AC-II and MBS cis-acting elements [36,37]. The BGLU family is a key gene in the synthesis of coumarin, and it has been reported that up-regulation of the transcription factor *MdMYB1r1* directly activates *MdBGLU40*, resulting in increased coumarin content in apples and increased resistance to pathogenic fungi [38]. In this paper, we analyzed the promoters of the apple MdBGLU family and identified promoters with AC-I, AC-II, and MBS cis-acting elements. These promoters were cloned and constructed into the pABAi vector for yeast one-hybrid experiments (Figure S5). The results showed that all three alternative spliced variants of *MdMYB6-like* could bind to the promoter of *MdBGLU H* (Figure 5), suggesting that *MdMYB6-like* could be used to respond to pathogen invasion by directly regulating the expression level of *MdBGLU H* to control the coumarin content.

## 3. Discussion

Diseases are a major factor limiting apple (*Malus domestica*) production [39]. Defending against infection is a very important issue during plant growth, but the disease defense mechanisms of woody plants such as apple have received less attention than those of traditional model plants such as the Arabidopsis thaliana (*A. thaliana*) and rice (*Oryza sativa*) [40,41]. Lignin is the main component of the secondary cell wall of plants, which can help to increase the structural strength of plant cells and plays an important role as a passive barrier against pathogen attack [42,43,44]. Previous studies have shown that the R2R3-MYB transcription factor family can be involved in the response to a variety of abiotic and biotic stresses by regulating key genes in the phenylalanine metabolic pathway. Heterologous expression of the R2R3-MYB transcription factor *CmMYB1* from chrysanthemum (*Chrysanthemum morifolium*) enhances lignin synthesis and inhibits flavonoid synthesis in Arabidopsis thaliana [45]. *PtrMYB152*, a R2R3-MYB family transcription factor from poplar (*Populus trichocarpa*), is a specific transcriptional activator for lignin biosynthesis during poplar wood formation [46]. *CfMYB4* and *CfMYB5* in Chinese cedar (*Cryptomeria fortunei*) can promote and inhibit lignin and cellulose accumulation, respectively, which not only highlights the regulatory function of CfMYBs in lignin content but also provides important insights into the development of genetic improvement strategies for Chinese cedar wood biomass [47]. However, few studies have focused on the involvement of the apple R2R3-MYB transcription factor family in apple lignin biosynthesis and its role in defending against pathogen infection.

Alternative splicing provides protein diversity during plant development and in response to abiotic and biotic stresses. A number of studies have shown that alternative splicing is involved in almost all biological processes, including signal transduction, energy transduction and secondary metabolite synthesis in higher animals and plants [48,49,50,51]. In practical research, alternative splicing events may produce splicing variants with low expression levels, occurring at particular times, which can make them relatively difficult to find, verify for authenticity and address other related issues. In this study, we used apple leaves cDNA as a template to clone three PCR products of 609 bp, 918 bp and 937 bp, which we named *MdMYB6-like-α*, *MdMYB6-like-β* and *MdMYB6-like-γ*. Structural analyses and genomic sequence comparisons revealed that both *MdMYB6-like-α* and *MdMYB6-like-β* isoforms undergo intron retention in variable splicing events. Both *MdMYB6-like-α* and *MdMYB6-like-β* isoforms undergo intron retention in variable splicing events. The occurrence of this phenomenon caused the forward shift of the stop codon, which led to the early termination of transcription and protein truncation. We performed RT-qPCR analysis of apple leaves at different times after infection with AAAP and found that the *MdMYB6-like-β* variant was significantly increased at 36 h post infection, and used this as the basis for subsequent studies. The other two isoforms also experienced increased expression levels at different times after pathogen infection. We also found that all three variants of *MdMYB6-like* are nuclear-localized transcription factors with no auto-activation activity, suggesting that the C-terminal truncation does affect the expression location and auto-activation activity of the gene. We also performed a validation of the existence of interactions between the variants and the results indicate that there are no interactions between their variants. We performed transient overexpression of the *MdMYB6-like* on ‘Gala’ leaves and found that overexpression of *MdMYB6-like-β* significantly increased the lignin content in ‘Gala’ leaves. Conversely, the *MdMYB6-like-α* isoform showed a significant decrease in lignin content, and *MdMYB6-like-γ* did not affect it significantly. Furthermore, only the overexpression of *MdMYB6-like-β* significantly increased the expression of *MdLac3*, which is involved in lignin synthesis, and this might be the reason for the increase in its lignin content. We used this as the basis for our experiments on the stable overexpression of *MdMYB6-like-β* in apple flesh callus and performed infection experiments on the positive lines with AAAP. The results showed that the OE-*MdMYB6-like-β* lines were significantly more resistant to AAAP compared to the control.

MYB transcription factors are common transcriptional regulators in the phenylpropanoid biosynthetic pathway [52,53]. Some MYBs act independently, but others interact with basic helix-loop-helix (bHLH) and WD40 proteins to form a protein complex called the MYB-bHLH-WD40 (MBW) complex [54]. In the previous study, the increase in lignin was also found in *PbMYB8*, the homologous gene of *MdMYB6-like* in ‘Dangshansuli’ pear, which is also in the Rosaceae family. Their experiment showed that *PbMYB8* can not only bind to the MBW complex comprising the bHLH and WD-repeat proteins but also directly regulate *CCoAOMT*. In addition, it is involved in regulating the expression of other genes involved in the phenylpropane metabolic pathway [32]. However, in our experiments, we found that both *MdMYB6-like* and its individual variants can directly bind to the promoter of *MdBGLU H*.

In conclusion, our results showed that there are three alternative spliced variants of the *MdMYB6-like* gene, and that the *MdMYB6-like-β* variant responds positively to pathogen infection, increasing the lignin content to reduce the effects of pathogen attack. This provides new ideas for breeding highly resistant apple cultivars. However, how *MdMYB6-like* regulates *MdBGLU H*, whether there is competitive inhibition between variants and whether its disease resistance mechanism is related to coumarin require further investigation.

## 4. Materials and Methods

### 4.1. Plant and Material

The ‘Starking Delicious’ and ‘Jonathan’ cultivars were grown at the Experimental Station of Nanjing Agricultural University, Jiangsu Province, China. The ‘Starking Delicious’ cultivar is susceptible to AAAP, while the ‘Jonathan’ cultivar is not. Healthy six-year-old apple trees were selected for the experiments, and the fully expanded fourth and fifth leaves on the new branches of the year were collected for the pathogen infection experiment and the determination of lignin content. Use absorbent skimmed cotton for water retention to prevent the leaves from losing water and drying out.

‘Gala’ is the apple tissue culture seedlings, which were cultured in vitro using Murashige and Skoog (MS) medium supplemented with 0.3 mg L^−1^ indole-3-acetic acid (IAA), 0.2 mg L^−1^ 6-benzyl amino purine(6-BA), and 0.1 mg L^−1^ gibberellic acid (GA) at 25 °C under 16-hlight/8 h dark cycles. Half of the MS medium, supplemented with 1.0 mg L^−1^ IAA and 0.4 mg L^−1^ indole-3-butyric acid (IBA), was used for rooting. When the leaves of the rooted seedlings were fully expanded, they were used for transient transfection experimental treatments.

The apple flesh callus tissue taken from ‘Orin’ apple fruits was cultured in vitro using Murashige and Skoog (MS) medium, supplemented with 1.5 mg L^−1^ 2,4-Dichlorophenoxyacetic acid (2,4-D) and 0.4 mg L^−1^ 6-benzyl amino purine(6-BA) at 25 °C under dark. The apple healing tissues were cultured on the medium for two weeks for subsequent experimental treatments.

Subcellular localization was conducted using *N. benthamiana* leaves. *N. benthamiana* seedlings were grown in a greenhouse at 24 °C under conditions of 16 h light/8 h dark cycles and used four weeks after germination. The seeds for the *N. benthamiana* came from the Fruit Tree Biotechnology Laboratory, College of Horticulture, Nanjing Agricultural University, Nanjing, China.

### 4.2. Pathogen Culture and Inoculation

The apple leaf spot disease pathogen (*Alternaria alternata* apple pathotype, AAAP) was gifted to us initially by the Northwest A&F University, and has since been preserved by our laboratory. The AAAP strain was cultured on potato dextrose agar (PDA) at 25 °C in the dark [55]. The strains that were cultured for 7 d and exhibited good activity were selected, and the most active mycelium at the edge was chosen as the pathogen, using a 5 mm picker to treat the leaves of the ‘Starking Delicious’ and ‘Jonathan’ cultivars. Three spots were inoculated on each side of the main veins of the leaves and empty PDA medium was used as a control treatment. The inoculated leaves were kept in incubation at 100% RH and 25 °C and then sampled at 0, 8, 12, 24, 36, and 72 h, respectively.

### 4.3. Gene Cloning and Sequence Analysis

Total RNA was extracted from all of plant material using an RNAprep pure Plant Kit (TianGen Biotech, Beijing, China) and cDNA was synthesized using a PrimeScript™ 1st Strand cDNA Synthesis Kit (Takara, Beijing, China). RT-PCR (reverse transcription PCR) was used to clone the transcripts of *MdMYB6-like* from plant material. Specific forward and reverse primers based on *MdMYB6-like* were used to amplify *MdMYB6-like* (Supplemental Table S1). The PCR reaction mixture comprised 10 μL 2× Hieff Canace Pre-Plus PCR Master Mix (Vazyme, Nanjing, China), 1 μL forward and reverse primers, 1 μL cDNA and 7 μL double distilled water. The PCR cycling parameters were as follows: pre-denaturation (98 °C, 3 min), 35 cycles (98 °C, 15 s; 55 °C, 15 s; 72 °C, 1 min) and a final extension (72 °C, 10 min). The PCR products were purified using an agarose gel extraction kit (Takara, Shanghai, China). The purified PCR products were cloned into a blunt-vector (Takara, Shanghai, China) and sequenced using M13 forward and reverse primers (Supplemental Table S1).

The obtained sequences were compared with template gene sequences (*MdMYB6-like* NCBI ID: XM_017336727.2) using DNAMAN. Sequences were aligned to the apple (malus domestica) genome to predict exons and introns using the online website GSDS2.0 (http://gsds.gao-lab.org/, accessed on 6 November 2022). Homologous protein sequences of *MdMYB6-like* and its variants were obtained using the blastp program available on the NCBI website (https://blast.ncbi.nlm.nih.gov/, accessed on 7 November 2022). The phylogenetic tree was constructed using the neighbor-joining method and visualized using MEGA6.0 software with a bootstrap value of 1000. The online website SMART (http://smart.embl-heidelberg.de/, accessed on 9 November 2022) was used for conserved domain analysis of putative protein sequences [56].

### 4.4. Subcellular Localization

The full-length coding regions of *MdMYB6-like-α*, *MdMYB6-like-β* and *MdMYB6-like-γ* were cloned and co-expressed with the green fluorescent protein (GFP) in the pCAMBIA1302-GFP vector between *Nco* I and *Spe* I sites and verified by RT-PCR using specific primers. The constructed vector was transferred into Agrobacterium strain GV3101, and injected into 5-week-old *N. benthamiana* leaves, which were subsequently cultured in the dark for 24 h and then in the photoperiod for 48 h. A LSM800 laser confocal fluorescence microscope (Carl Zeiss Jena, Jena, Germany) was then used to observe GFP fluorescence of *MdMYB6-like-α*, *MdMYB6-like-β* and *MdMYB6-like-γ*. *N. benthamiana* leaves that were injected with the empty pCAMBIA1302 plasmid were used as a positive control. Leaves were treated with 4′,6-diamidino-2-phenylindole (DAPI) to stain and identify the nucleus. Primer details are provided in Supplemental Table S1.

### 4.5. Yeast Two-Hybrid Assays

The pGADT7 [containing transcriptional activation domain (AD)] and pGBKT7 [containing DNA-binding domain (BD)] vectors were digested with restriction endonucleases for use in the Y2H assays. The pGADT7 used *Eco* R I and *Bam* H I as the enzymatic sites, while pGBKT7 selected *Sal* I and *Eco* R I, respectively. The *MdMYB6-like-α*, *MdMYB6-like-β* and *MdMYB6-like-γ* sequences were inserted into pGADT7 and pGBKT7, respectively. The constructed plasmids were transformed together in different combinations into the yeast strain Y2HGold using the PEG/LiAc method. Auto-activation activity of *MdMYB6-like-α*, *MdMYB6-like-β* and *MdMYB6-like-γ* was assessed. The yeast colony PCR was used to identify which yeast culture on double-deficient medium (SD-Leu/-Trp) are positive transformants. Positive yeast colonies were cultured in liquid medium and transferred to a quadruple-deficient medium (SD-Leu/-Trp/-His/-Ade) to be screened again for interactions. X-α-Gal was used to assess β-‘Gala’ ctosidase activity to further confirm positive interactions.

### 4.6. Transient Transformation of Apple Leaves

The transcripts of *MdMYB6-like-α*, *MdMYB6-like-β* and *MdMYB6-like-γ* were cloned and inserted into the pCAMBIA2300 vector between the restriction enzyme sites *Sal* I and *Xba* I to construct the plant expression vectors pCAMBIA2300-*MdMYB6-like-α*, pCAMBIA2300-*MdMYB6-like-β* and pCAMBIA2300-*MdMYB6-like-γ*, respectively. The constructs were transformed into Agrobacterium tumefaciens GV3101 using the freeze–thaw method and cryopreserved at −80 °C. Positive strains were resuspended in infiltration buffer (10 mmol/L MgCl_2_ and 10 mmol/L MES to an OD_600_ of 0.8; acetosyringone was added before use) and incubated at 28 °C in the dark for 4 h before the next one-step experiment [57]. Subsequently, the leaves of ‘Gala’ rooted seedlings were immersed in the resuspension solution and vacuumed until the surface of the leaves was slightly translucent. The empty vector pCAMBIA2300 was used as a control treatment. After dipping, the leaves were incubated at 25–28 °C and 60% to 70% relative humidity until the infestation solution was completely absorbed. The samples were collected and set aside at −80 °C. The experiment was repeated three times.

### 4.7. Stable Transformation of Apple Flesh Callus

The positive Agrobacterium successfully transferred to the constructed plasmid was resuspended in MS_0_ media (100 μmol/L acetosyringone + MS) and incubated at 28 °C without light for 30 min prior to use for infiltration. The apple flesh callus was crushed into the infiltration buffer and incubated with shaking at 28 °C for 15 min. The empty vector pCAMBIA2300 was used as a control treatment. The flesh callus were filtered out with gauze, blotted dry with filter paper and spread on screening medium (MS medium plus 300 μmol/L kanamycin and 2 mol/L Timentin), cultured at 28 °C in the dark for one month. Screening of positive strains was conducted using the β-glucuronidase (GUS) stain.

### 4.8. Total Lignin Extraction and Quantification

The phenolic hydroxyl group in lignin has a characteristic absorption peak at 280 nm after acetylation, and the high absorbance value at 280 nm is positively correlated with the lignin content [58]. Experiments were performed with the same infestation solution as that used for transient transformation of apple leaves. Roots of ‘Gala’ apple rooted seedlings were wrapped in plastic wrap along with the culture medium, and then the above ground parts were completely immersed in the infiltration solution for transient transformation experiments. The infested seedlings were cultured at 28 °C, in the dark, for three days, after which they continued to be cultured under 16 h light/8 h dark cycles for three days. Healthy leaves were selected for sampling and the leaf samples were baked in an oven at 80 °C until the leaves were of constant weight, ground and sieved. Additionally, lignin concentration after transient transformation of apple leaves was determined by a lignin assay kits (Solarbio, Beijing, China) according to the instructions provided by the manufacturer.

### 4.9. Real-Time Quantitative PCR Analysis

RT-qPCR was performed to detect the expression of *MdMYB6-like* and its alternative spliced variants in apple leaves of ‘Starking Delicious’ and ‘Jonathan’ cultivars at different time points after inoculation. RT-qPCR analysis was conducted to examine lignin biosynthesis pathway gene expression in transiently transformed ‘Gala’ leaves. Gene-specific primers were designed using the online website NCBI (https://www.ncbi.nlm.nih.gov/, accessed on 12 April 2023). The specificity of the primers was tested by determining standard curves for each gene. RT-qPCR was performed using an Applied Biosystems QuantStudio^®^ 5 Real-Time PCR System (Thermo Scientific, Shanghai, China). The PCR reaction mixture consisted of 10 μL SYBR Premix Ex Taq (Takara, Beijing, China), 0.8 μL forward and reverse primers, 0.4 μL ROX, 1 μL cDNA and 7 μL double distilled water. The PCR cycling parameters included denaturation (95 °C, 30 s), followed by 40 cycles (95 °C, 5 s; 60 °C, 34 s), with signal acquisition (72 °C, 30 s). Gene transcript levels were normalized using an *β-tublin* (NCBI ID: XM_008378370.3). Relative expression values were calculated using the 2^−ΔΔCt^ method. Three biological and technical replicates were used in the RT-qPCR analysis of each sample [59]. All primers used in the RT-qPCR analyses are listed in Supplemental Table S1.

### 4.10. Yeast One-Hybrid Assays

The NCBI website was used to locate the promoters of the target genes. We used the PlantCARE website (http://bioinformatics.psb.ugent.be/, accessed on 12 April 2023) for promoter sequence analysis [60]. *MdBGLU H* (NCBI ID: XM_029099134) promoter sequences were cloned into the pAbAi vector for Y1H assays (Supplemental Table S1). The constructed vector plasmids were digested by *Bst* B I, and the linearized products were transferred into yeast strain Y1HGold and screened on a single-deficient (SD-Ura) medium after confirming the integration of decoy sequences by colony PCR. The AD-*MdMYB6-like-α*, AD-*MdMYB6-like-β*, AD-*MdMYB6-like-γ* and empty pGADT7 vector plasmids from the Y2H assays were transferred into the Y1HGold (Bait-AbAi) strains. Screening of positive strains was performed on an SD/-Leu medium containing different concentrations of ABA to verify the interaction between promoters and proteins. Empty pGADT7 vector was used as the control.

## 5. Conclusions

We found that *MdMYB6-like* in apple contains three alternative spliced variants, which we named *MdMYB6-like-α*, *MdMYB6-like-β* and *MdMYB6-like-γ*. Then we found that all three variants of the *MdMYB6-like* are nuclear-localized transcription factors with no auto-activation activity. Under normal growth conditions, *MdMYB6-like-α*, *MdMYB6-like-β* and *MdMYB6-like-γ* cannot form homodimers or heterodimers, respectively. In the following experiments, we show that *MdMYB6-like-β* can significantly increase the lignin content and improve the resistance of apple flesh callus to AAAP. Finally, we found that both *MdMYB6-like* and its variants can bind to the promoter of *MdBGLU H*. These could be the main factors of *MdMYB6-like* affecting disease resistance in apple.

In summary, we concluded that *MdMYB6-like* is a transcription factor with potential for disease resistance, which may theoretically support the future breeding of apple cultivars with higher resistance to pathogen invasion. Due to the high level of apple leaf spot disease on leaves, in this paper, the stabilized transformed material is the callus. Therefore, the results are limited to the improvement of disease resistance in callus by *MdMYB6-like-β* isoforms. Additionally, the level of disease resistance on leaves and the whole of the plant need to be further investigated. Furthermore, the specific molecular mechanism of *MdMYB6-like* regulation needs to be further investigated.

## Data Availability

Data is contained within the article and Supplementary Materials.

## Supplementary Materials

The following supporting information can be downloaded at: https://www.mdpi.com/article/10.3390/ijms25084353/s1.
