# Supplementary material for: Molecular Mechanism of Resistance to Alternaria alternata Apple Pathotype in Apple by Alternative Splicing of Transcription Factor MdMYB6-like
Source: Int J Mol Sci. 2024 Apr 15;25(8):4353. doi: 10.3390/ijms25084353 (PMC11050356; doi:10.3390/ijms25084353)
Supplement: Supplementary file 1 [file ijms-25-04353-s001.zip › Figure S2/Figure S2.pdf]

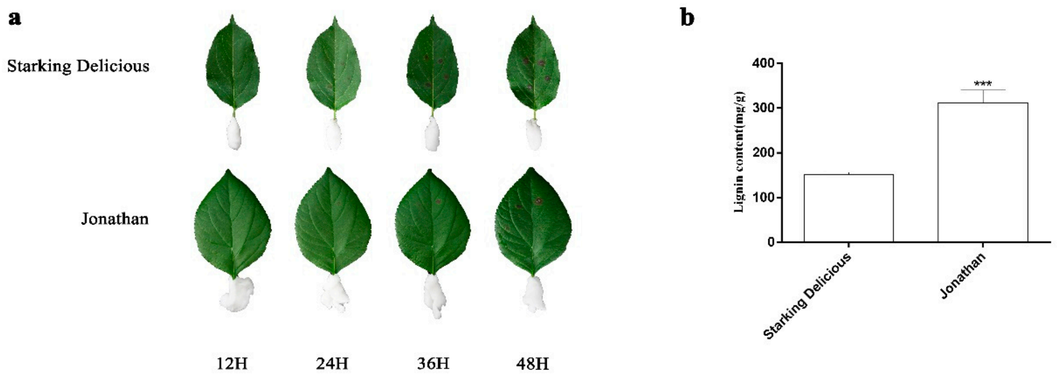

Figure S2. Differential performance of apple cultivars with different resistance to AAAP. (a) Three plaques were inoculated on each side of the main vein of the leaves of 'Starking Delicious' and 'Jonathan'. Photographs were taken at different times (12, 24, 36, 48H) after inoculation. (b) 'Starking Delicious' and 'Jonathan' leaves of the same weekly age were taken and dried at 80°C in an oven. Lignin content was determined by taking 3mg of dried leaf samples. Error bars represent the SDs from three biological replicates. The level of significant difference is indicated by * which significant differences at $p < 0.05$.
