# Supplementary material for: Molecular Mechanism of Resistance to Alternaria alternata Apple Pathotype in Apple by Alternative Splicing of Transcription Factor MdMYB6-like
Source: Int J Mol Sci. 2024 Apr 15;25(8):4353. doi: 10.3390/ijms25084353 (PMC11050356; doi:10.3390/ijms25084353)
Supplement: Supplementary file 1 [file ijms-25-04353-s001.zip › Figure S3/Figure S3.pdf]

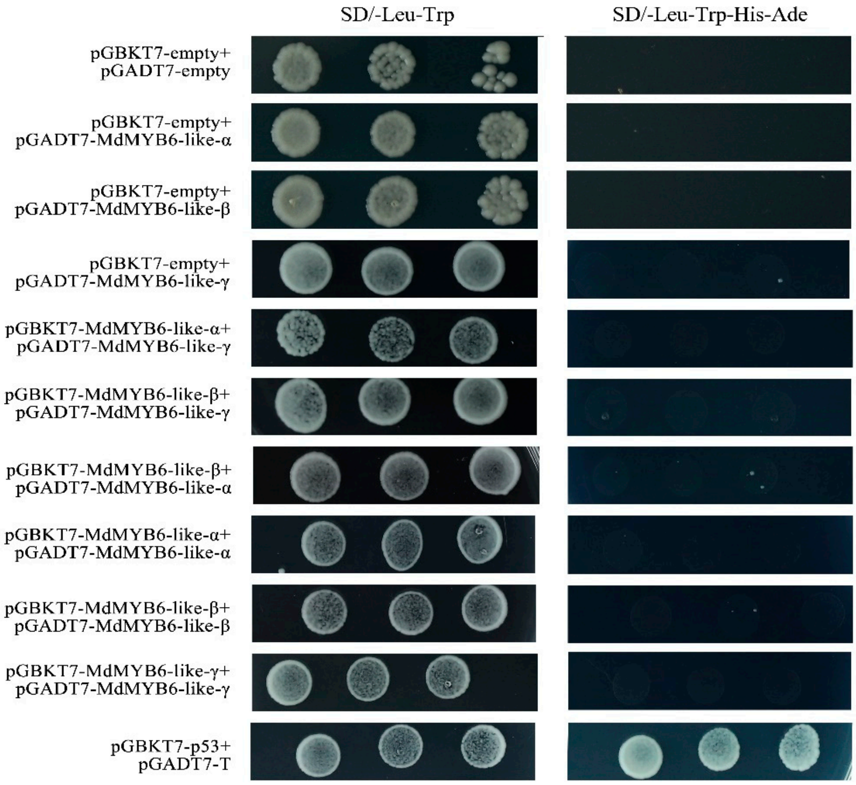

Figure S3. Yeast two-hybrid of binding of MdMYB6-like and its alternative spliced variants to form homologous and heterologous complexes. Transformed the constructed plasmid into the AH109 yeast strain in different combinations. Co-transformation of empty pGADT7 and pGBKT7 vector was used as a negative control, and co-transformation of pGBKT7-p53 and pGADT7-T vector was used as a positive control. Three repetitions for each combination.
