# Supplementary material for: Molecular Mechanism of Resistance to Alternaria alternata Apple Pathotype in Apple by Alternative Splicing of Transcription Factor MdMYB6-like
Source: Int J Mol Sci. 2024 Apr 15;25(8):4353. doi: 10.3390/ijms25084353 (PMC11050356; doi:10.3390/ijms25084353)
Supplement: Supplementary file 1 [file ijms-25-04353-s001.zip › Figure S4/Figure S4.pdf]

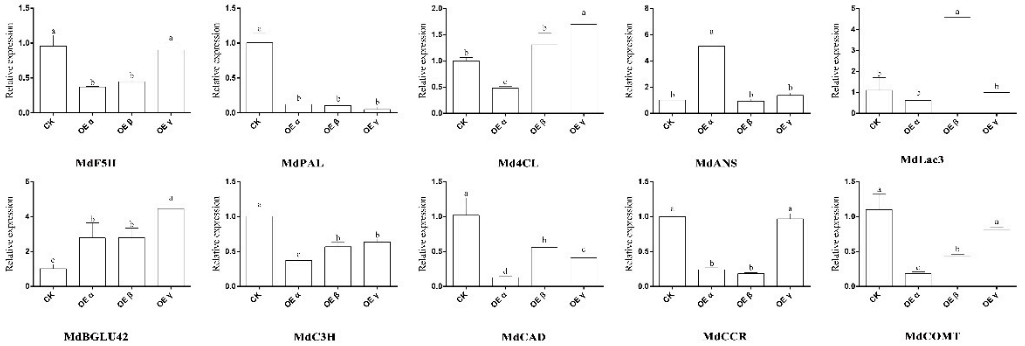

Figure S4: Using RT-qPCR analysis to expression level changes of lignin synthesis-related genes after transient overexpression of MdMYB6-like and its alternative spliced variants. The genes involved in the analysis included MdF5H (NCBI Reference Sequence: XM_008374531.3), MdPAL (NCBI Reference Sequence: XM_008389362.3), Md4CL (NCBI Reference Sequence: XM_029091143.1), MdANS (NCBI Reference Sequence: NM_001328948.1), MdLAC3 (NCBI Reference Sequence: XM_008386485.3), MdBGLU42 (NCBI Reference Sequence: XM_008376601.3), MdC3H (NCBI Reference Sequence: XM_008380681.3), MdCAD (NCBI Reference Sequence: NM_001328793.1), MdCCR (NCBI Reference Sequence: XM_008377320.3), MdCOMT (NCBI Reference Sequence: XM_008368176.3). Error bars represent the SDs from three biological replicates. Lowercase letters represent significant differences at $p < 0.05$ (Tukey's HSD test).
