# Supplementary material for: Molecular Mechanism of Resistance to Alternaria alternata Apple Pathotype in Apple by Alternative Splicing of Transcription Factor MdMYB6-like
Source: Int J Mol Sci. 2024 Apr 15;25(8):4353. doi: 10.3390/ijms25084353 (PMC11050356; doi:10.3390/ijms25084353)
Supplement: Supplementary file 1 [file ijms-25-04353-s001.zip › Figure S5/Figure.S5.pdf]

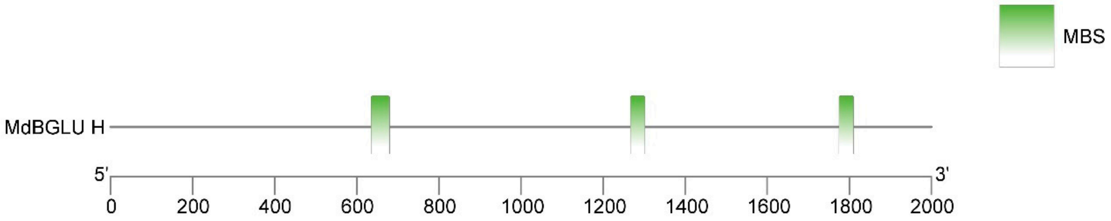

Figure S5. Visualization of the promoter of MdBGLU H. The cis-acting element MBS is marked with a green box. The location of the elements on the promoter is shown in the figure.
