# Supplementary figures and images for: Molecular Mechanism of Resistance to Alternaria alternata Apple Pathotype in Apple by Alternative Splicing of Transcription Factor MdMYB6-like
Source: Int J Mol Sci. 2024 Apr 15;25(8):4353. doi: 10.3390/ijms25084353 (PMC11050356; doi:10.3390/ijms25084353)

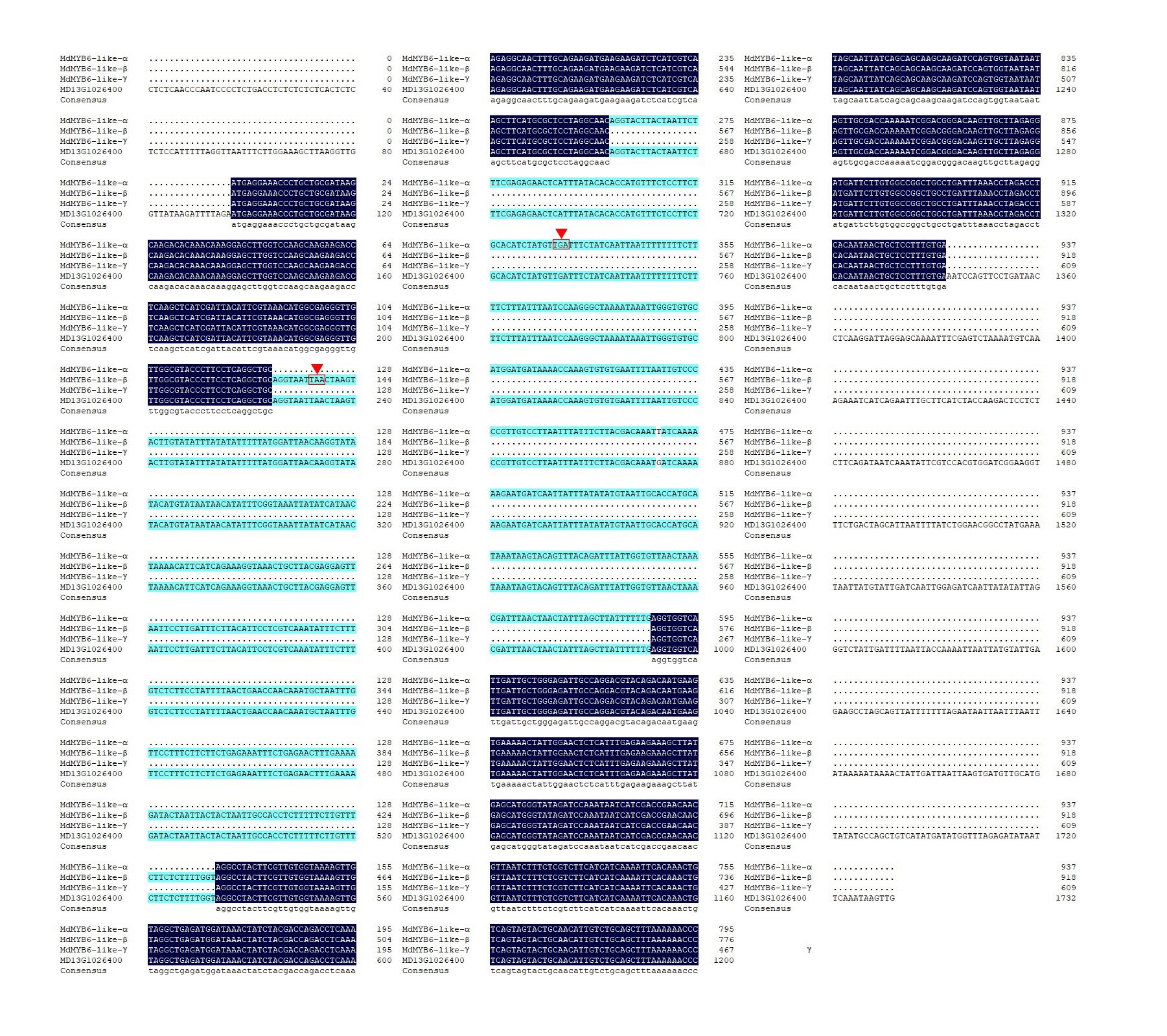

Supplement: Supplementary file 1 [file ijms-25-04353-s001.zip › Figure S1/Figure S1.jpg]

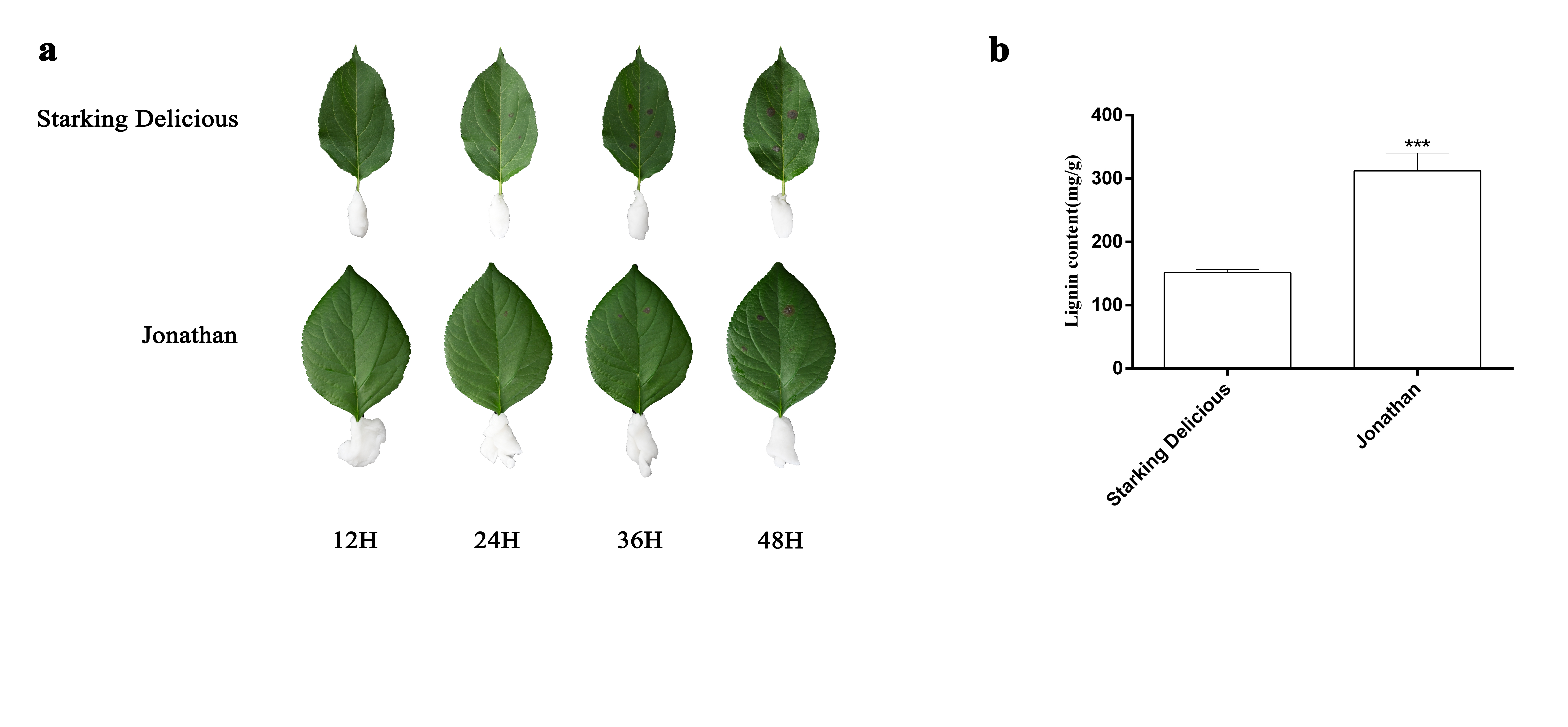

Supplement: Supplementary file 1 [file ijms-25-04353-s001.zip › Figure S2/Figure S2.jpg]

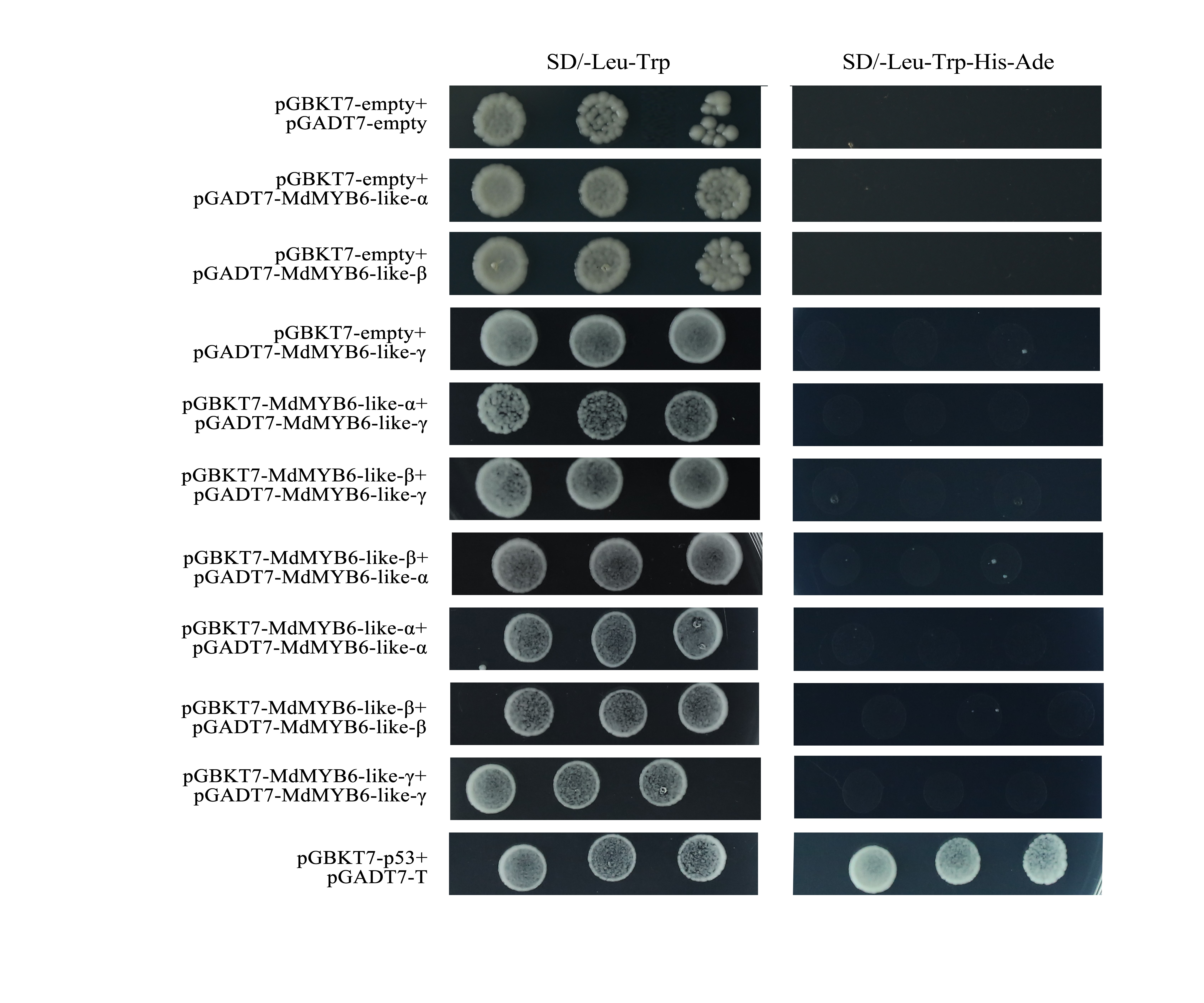

Supplement: Supplementary file 1 [file ijms-25-04353-s001.zip › Figure S3/Figure S3.jpg]

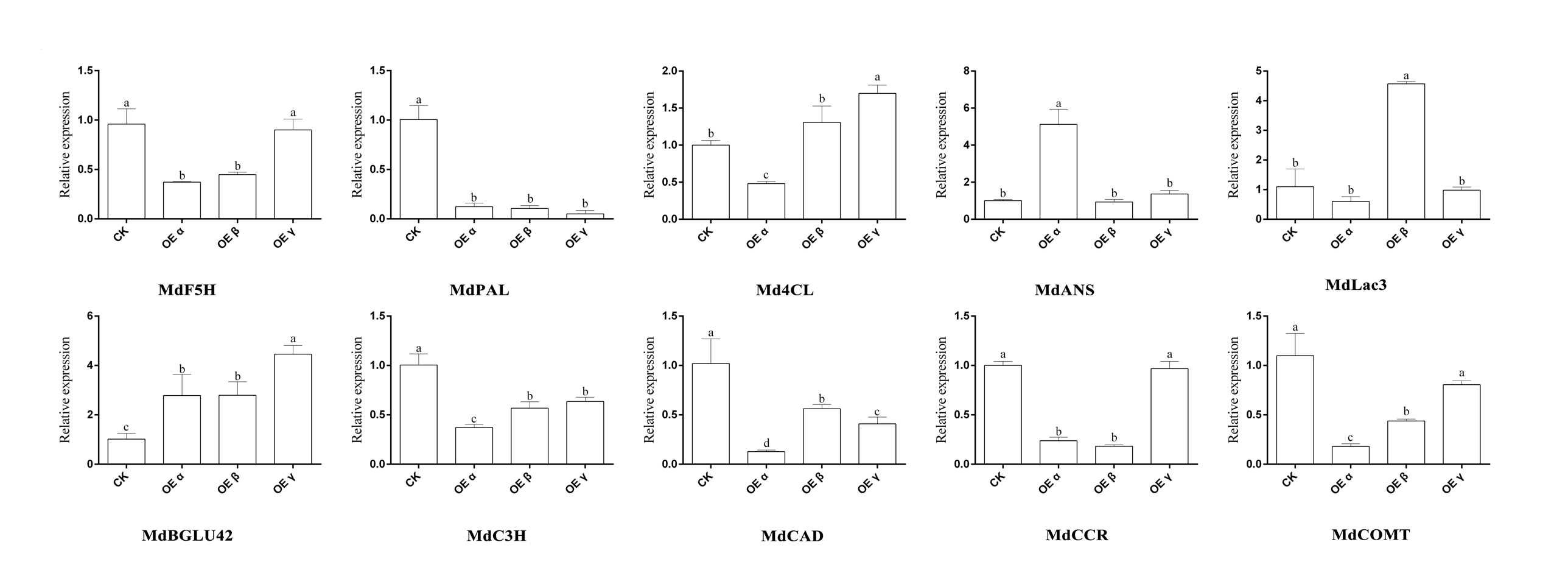

Supplement: Supplementary file 1 [file ijms-25-04353-s001.zip › Figure S4/Figure S4.png]

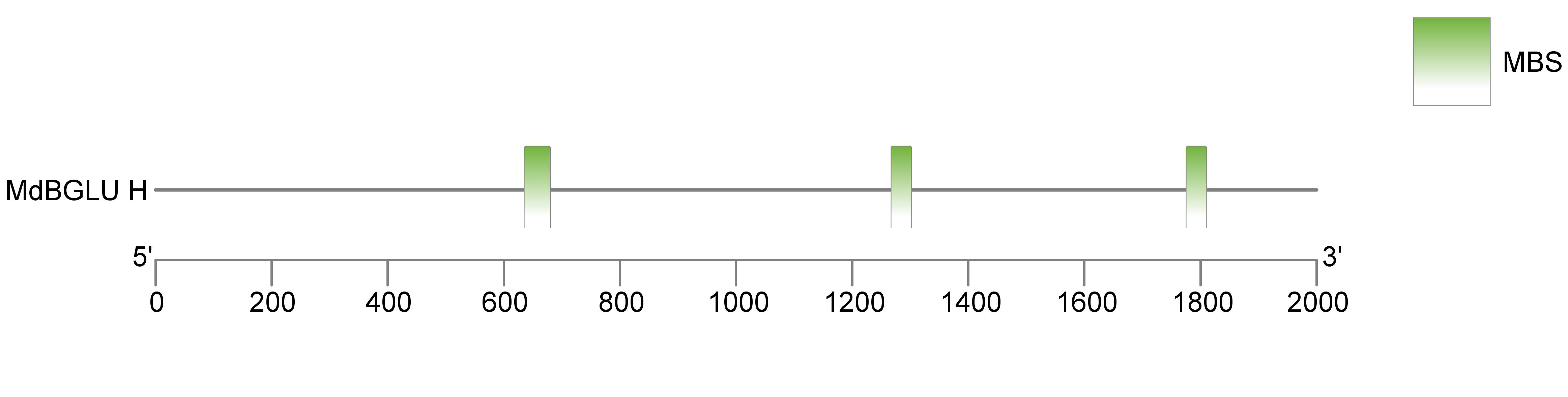

Supplement: Supplementary file 1 [file ijms-25-04353-s001.zip › Figure S5/Figure S5.jpg]
