# Supplementary material for: Molecular Mechanism of Resistance to Alternaria alternata Apple Pathotype in Apple by Alternative Splicing of Transcription Factor MdMYB6-like
Source: Int J Mol Sci. 2024 Apr 15;25(8):4353. doi: 10.3390/ijms25084353 (PMC11050356; doi:10.3390/ijms25084353)
Supplement: Supplementary file 1 [file Figure S1.pdf › Figure S1/Figure S1.pdf]

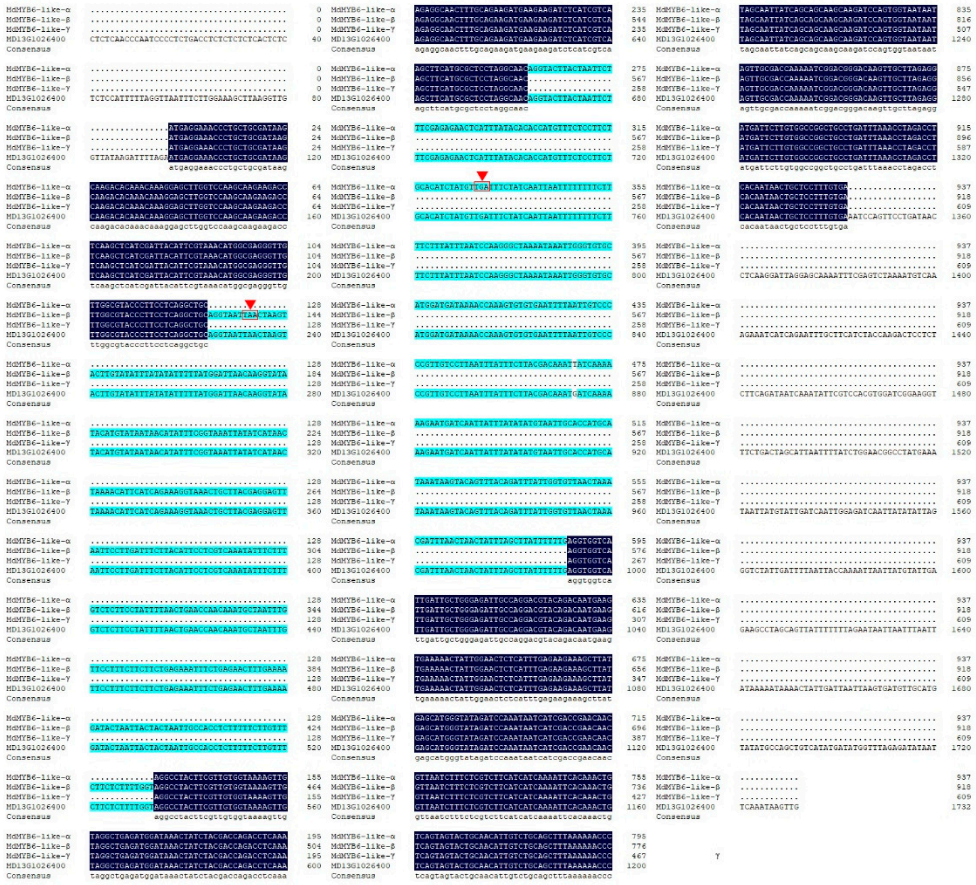

Figure S1. Three alternative splicing sequence alignments of MdMYB6-like in malus domestica. Nucleotide sequence alignment of *MdMYB6-like-α*, *MdMYB6-like-β* and *MdMYB6-like-γ*. The red triangles and boxes indicate a premature termination codon of the two variants, respective.
